# Serendipitous Meta-Transcriptomics: The Fungal Community of Norway Spruce (*Picea abies*)

**DOI:** 10.1371/journal.pone.0139080

**Published:** 2015-09-28

**Authors:** Nicolas Delhomme, Görel Sundström, Neda Zamani, Henrik Lantz, Yao-Cheng Lin, Torgeir R. Hvidsten, Marc P. Höppner, Patric Jern, Yves Van de Peer, Joakim Lundeberg, Manfred G. Grabherr, Nathaniel R. Street

**Affiliations:** 1 Department of Plant Physiology, Umeå Plant Science Centre, Umeå University, Umeå, Sweden; 2 Science for Life Laboratory, Department of Medical Biochemistry and Microbiology, Uppsala University, Uppsala, Sweden; 3 Bioinformatics Infrastructure for Life Sciences (BILS), Uppsala, Sweden; 4 Department of Plant Systems Biology (VIB) and Department of Plant Biotechnology and Bioinformatics (Ghent University), Ghent, Belgium; 5 Department of Chemistry, Biotechnology and Food Science, Norwegian University of Life Sciences, Ås, Norway; 6 Genomics Research Institute, University of Pretoria, Pretoria, South Africa; 7 School of Biotechnology, Science for Life Laboratory, KTH Royal Institute of Technology, Solna, Sweden; University of Innsbruck, AUSTRIA

## Abstract

After performing *de novo* transcript assembly of >1 billion RNA-Sequencing reads obtained from 22 samples of different Norway spruce (*Picea abies*) tissues that were not surface sterilized, we found that assembled sequences captured a mix of plant, lichen, and fungal transcripts. The latter were likely expressed by endophytic and epiphytic symbionts, indicating that these organisms were present, alive, and metabolically active. Here, we show that these serendipitously sequenced transcripts need not be considered merely as contamination, as is common, but that they provide insight into the plant’s phyllosphere. Notably, we could classify these transcripts as originating predominantly from Dothideomycetes and Leotiomycetes species, with functional annotation of gene families indicating active growth and metabolism, with particular regards to glucose intake and processing, as well as gene regulation.

## Introduction

To aid gene prediction within the Norway spruce genome project [1], we sequenced more than half a billion paired end reads (2 x 100 bps) using the oligo(dT) protocol from samples collected from various tree tissues. All samples (see S1 Fig, detailed in S1 Table) were collected with full permission from mature Norway spruce individuals within a national, clonal archive of breeding material maintained by Skogforsk (the Forestry Research Institute of Sweden). Since the samples were not surface sterilized, this experiment inadvertently allowed us to gain a glimpse into the complex phyllospheric eukaryotic community associated with mature *P*. *abies* trees, including endophytes, epiphytes, lichen and other organisms.

## Material and Methods

### Sample collection and sequencing

Samples were collected from various tree tissue types throughout the growing season, as detailed in S1 Fig and Supplementary Material Section 2 of Nystedt *et al*. [1]. Briefly, with the exception of Sample 10 (Z3001TR10), all samples were collected during the 2010 growing season from a single 43 year-old clonal replicate of *Picea abies* clone ‘Z4006’, the genotype used for the Norway spruce genome project [1], growing at the Skogforsk tree archive, Sävar, Umeå in north Sweden (63°18’N/15°59’E). Sample 10 was collected from clone ‘Z3001’ growing at the same location and of the same age. Samples were not surface sterilised and therefore all endophytes, epiphytes, lichen and other organisms that were tightly bound to the samples were also included (*i*.*e*. the phyllosphere).

Sequence data were generated using standard Illumina mRNA sequencing protocols and kits (paired end 2 x 100 bps), generating >23M Paired-End reads per library on average. Briefly, RNA was extracted using a modified version of the CTAB method [2] and further purified using an RNeasy Mini Kit (Qiagen, Hilden, Germany) according to the manufacturer’s protocol. Total RNA preparations were sent to the Beijing Genome Institute (BGI, Shenzhen, China) for RNA-Seq sequencing using standard Illumina protocols and kits (TruSeq SBS KIT-HS v3, FC-401-3001; TruSeq PE Cluster Kit v3, PE-401-3001) on the Illumina HiSeq 2000 platform. The sequencing protocol involved DNase 1 digestion of total RNA, mRNA isolation with oligo(dT) beads, mRNA fragmentation, first and second strand cDNA synthesis, end-repair, A-tailing, bar-coded adapter ligation and PCR amplification and all samples yielded >20 million paired-end reads (min 21,961,993 max 24,830,162). It is important to note that the sequencing library preparation protocol utilised oligo(dT) beads to enrich for poly-adenylated mRNAs and, as such, prokaryotic transcripts were not expected to be represented.

### Bioinformatics processing

A schematic overview of the bioinformatics pipeline used is outlined in S2 Fig Starting with the RNA-Sequencing reads, we performed a joint assembly of all samples and all organisms (left column) to provide a broad scale analysis and characterisation of the data, including abundance estimates for fungal transcripts. To provide a taxonomic context for fungal transcripts and to analyse fungal nuclear gene expression, we applied GC-content filters and assembled samples with detectable amounts of fungal RNA separately (right column). Details are described below.

### Joint *de novo* transcript assembly

An initial *de novo* transcriptome assembly was performed using Trinity [3] (release 2012-06-08), where sequencing reads from the 22 libraries were combined (totalling 522,400,141 read pairs) and assembled using the settings—seqType fq—min_kmer_cov 2—group_pairs_distance 500—path_reinforcement_distance = 50—bfly_opts—edge-thr = 0.20. The percentage Guanine—Cytosine (GC) nucleotide content of transcripts was calculated as the number of “C” and “G” nucleotide occurrences divided by transcript length with these values then multiplied by a 100 and rounded to 2 decimal places.

A putative taxonomic origin for each assembled transcript was assigned through alignment to the Uniref90 protein database [4] using blastx and selecting the top ranking alignment to known proteins, as indicated by the blastx e-value (at a cut-off of e < 10^−3^). All assembled transcripts were additionally aligned to the *P*. *abies* 1.0 genome assembly using GMAP [5] as described in Nystedt *et al*. [1]. Transcripts were considered to have a valid alignment if 99% of the GMAP alignment covered >80% of the transcript with >90% identity. Transcripts that had no taxonomic assignments from protein database alignments but had valid genome alignments were re-classified as originating from spruce. As detailed in Nystedt *et al*. [1], the available genome sequence was filtered to remove potential contaminant scaffolds using criteria similar to those described here for filtering non-spruce transcripts.

Expression quantification values based on alignment of RNA-Seq reads, calculated as Fragments Per Kilobase per Million mapped reads (FPKM), were assigned to each transcript and for each sample, as computed using RSEM applied with default parameters [6]. Reads were aligned to assembled transcripts using the Trinity alignReads.pl support script with default settings [7]. To indicate the degree of expression specificity across samples using log_2_(FPKM) expression values, we used the tau score [8]. Prior to the tau score calculation, FPKM values <1 were set to log_2_(FPKM) = 0 and transcripts with log_2_(FPKM) = 0 in all 22 samples were removed. Then with *a*_*ij*_ being the average expression of gene *i* in tissue *j*, the tissue specificity of gene *i* is given by

$$\tau_{i}=\frac{1}{n-1}\sum_{j=1}^{n}(1-\frac{a_{ij}}{\underset{j}{\text{max}}{(a}_{ij})})$$

where *n* is the number of tissues. Hence tissue specificity is calculated relative to the tissue with the highest mean expression *e*.*g*. a tau score of 0.8 indicates that the mean expression in other tissues is 20% of the expression in the tissue with the highest mean expression.

The obtained FPKM values were used to perform a Principal Component Analysis (PCA), using the prcomp function in R (version 3.0.2).

### GC content and expression identify *bona fide* nuclear fungal genes

To enrich for fungal nuclear protein coding sequences independent of known genes, we utilised differences in GC content and sample specificity (*i*.*e*. breadth of expression) between spruce and fungi. We selected RNA-Seq read pairs at 46% GC or higher, and re-assembled those separately for 10 libraries using *Trinity* with default parameters. The resulting transcript assemblies had a 20% N50 increase in transcript lengths compared to the combined initial assembly. To eliminate remaining spruce sequences, for which the tau scores indicated that they tended to be expressed in multiple tissues, we filtered out transcripts that were 100% identical over at least 100 nucleotides in at least five samples.

We exhaustively aligned the 59,260 identified percent GC and expression breadth filtered sequences against each other using the highly sensitive alignment program *Satsuma* [9], requiring overlaps of 200 nucleotides or more, at sequence similarity of >52%. We then grouped the sequences by agglomerative hierarchical clustering, using 1 minus the identity as the pairwise distance. To assign putative function to each gene cluster we aligned the longest sequence per cluster to the NCBI *nr* protein database using Blast2GO (http://www.blast2go.com/b2ghome). Blast2GO results were used to retrieve functional annotations and were further filtered for clusters where the longest sequence had top alignment hits to Viridiplantae (40% of the clusters), including Chlorophyta, which are similar in GC content to Fungi. Phylogenetic analyses were performed using the phylogeny.fr pipeline [10], using default parameters for multiple sequence alignment (maximum 16 iterations), computing maximum likelihood phylogenies and using the SH-like Approximate Likelihood-Ratio test to estimate branch support values. Visualisation was performed using the FigTree software (http://tree.bio.ed.ac.uk/software/figtree/). To build phylogenies, we selected reference sequences based on top BLAST alignment scores, as well as functional annotations other than ‘uncharacterized protein’. For quantification, we mapped all reads to the clustered assemblies, computed FPKM values using RSEM [6], and summed expression over the transcript sequences within clusters.

### Data Accessibility

Raw RNA-Seq data is available from the European Nucleotide Archive under the accession number ERP002475. Transcript assemblies, all transcript subsets described and meta-analyses results are available from ftp://plantgenie.org/Publications/Delhomme2015 and http://dx.doi.org/10.5061/dryad.b1500. Supplementary information is available at PLoS One’s website.

## Results and Discussion

To obtain an initial global assessment of transcript diversity, we first performed a *de novo* assembly of the combined set using *Trinity* [3]. The assembly comprised 352,575 transcripts with an N50 size of 683 nucleotides. Less than half (140,586) of those sequences showed significant (E < 10^−8^) sequence similarity to known proteins in the UniRef90 protein database. However, only 27.6% were classified as Viridiplantae, while 61.2% were assigned to the fungal kingdom, representing essentially ascomycetes and basidiomycetes (see S3 and S4 Figs). The majority of the remaining 11.2% had highest similarity to Bacteria and Metazoa.

**Fig 1:**
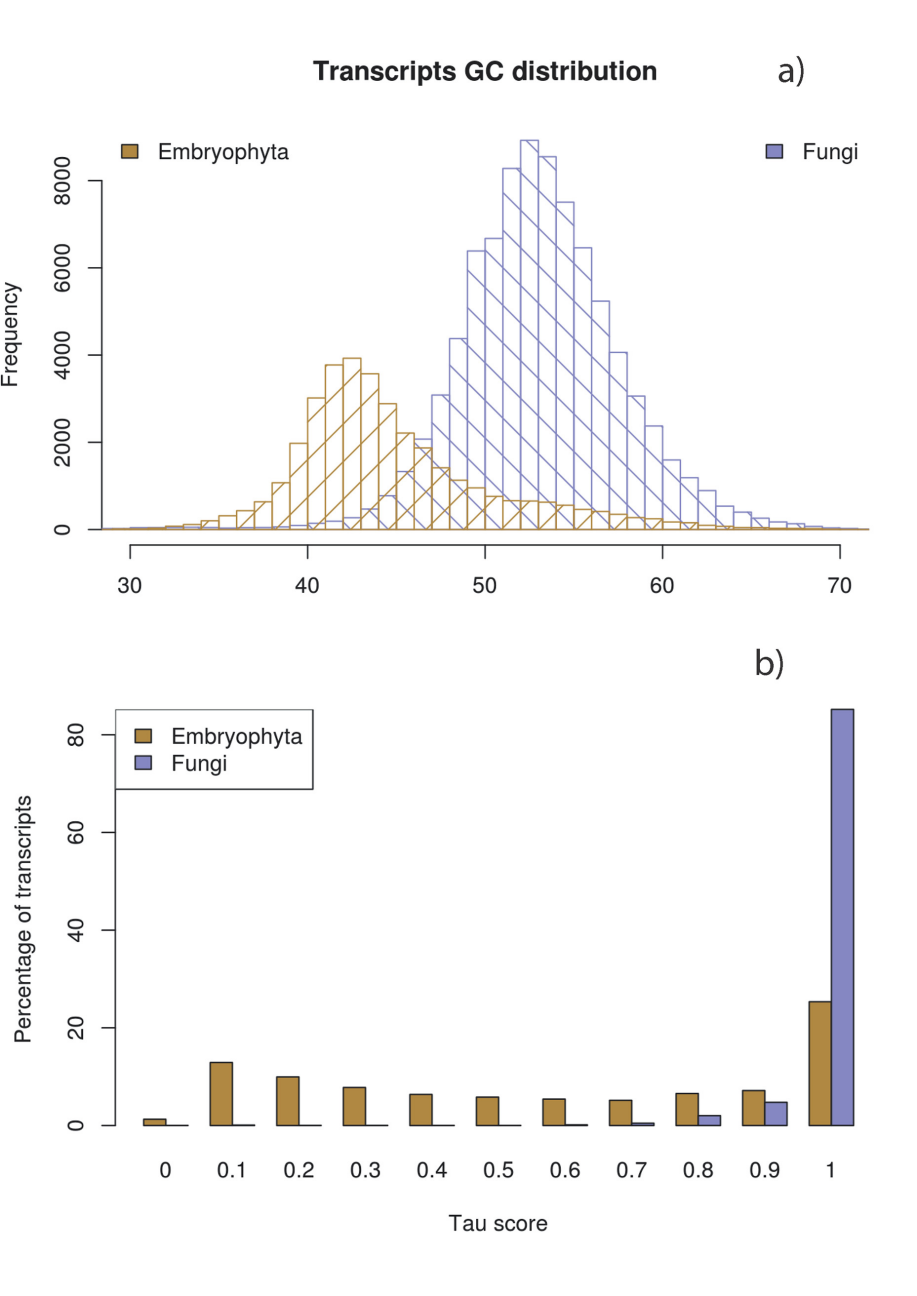
Two-dimensional distribution of expression and GC content differentiates fungal and plant transcripts. **(a)** Percentage GC frequency of transcripts annotated as Embryophyta (brown) or fungi (purple) on the basis of maximum sequence similarity to the UniRef90 database. **(b)** Frequency distribution of tau score calculated using FPKM expression values for plant (brown) and fungal (blue) transcripts in all 22 RNA sequencing samples. The tau score ranges from 1 for complete specificity to 0 for equal expression in all samples. The Embryophyta tau score distribution is significantly different from that of the fungi (Welch t-test p-value << 0.001)**.**

We identified striking differences between plant and fungal transcripts: plant sequences were on average longer (N50: 1,823 *vs* 453bp), exhibited higher total FPKM expression values (79.3M *vs*. 0.7M), and were lower in average GC content (44.5%, *vs*. 53.1%, Fig 1A). Transcripts with highest sequence similarity to Chlorophyta, a division of green algae including lichens, were similar to fungal transcripts both in expression values and GC content. In addition, the expression specificity across samples, as measured by the tau score [8] was lower for plants (average tau score = 0.74), indicating that many genes were expressed in multiple tissues or samples, while fungal transcripts exhibited almost complete specificity (average tau score = 0.99, Fig 1B). Moreover, fungal expression was largely confined to needle and bud samples and mostly absent from other parts of the tree.

Alignments of transcript sequences without known protein homology to the spruce genome (using GMAP [5]) resulted in a similar picture: for the 122,571 aligned sequences, both the tau scores and GC content characteristics largely followed transcripts with homology to plant proteins (S5 Fig), while transcripts without alignment (89,418) were similar to those classified as fungal by protein homology (S5 Fig).

Utilizing differences in GC content and expression patterns, we next generated an improved set of fungal transcripts by assembling 10 selected needle, bud, and vegetative shoot samples individually, using only read pairs at an average GC content of 46% or higher. The resulting sequences were 20% (N50) longer than the initial assembly. We next pooled homologous (both orthologous and paralogous) genes by nucleotide similarity (see Methods) into 1,127 clusters containing four or more members, and assigned putative function to each gene cluster using Blast2GO. A phylogeny built using the highly conserved translation elongation factor *(TEF)* grouped transcripts with known Eurotiomycetes, Sordariomycetes, and Dothideomycetes reference sequences (S6 Fig), in particular with the Dothideomycete order Pleosporales (*Lophiotrema*, *Hysteropatella*, and *Massariosphaeria*), as well as with the Capnodiales (*Devriesia*). One sequence grouped with Leotiomycetes, and two sequences each were placed close to Eurotiomycetes, Sordariomycetes, and a group of Ustilaginomycetes in the division Basidiomycetes. We repeated the analysis for a number of additional gene families (among them phosphoenolpyruvate carboxykinase (*PEPCK*); NADP-dependent medium chain alcohol dehydrogenase (*NMCA*); and beta lactamase) resulting in similar phylogenetic relationships (S6 Fig), suggesting that the class Dothideomycetes were the most common in these datasets, but also that the taxonomic breadth could be large, possibly indicating sample-specific colonization patterns.

**Fig 2:**
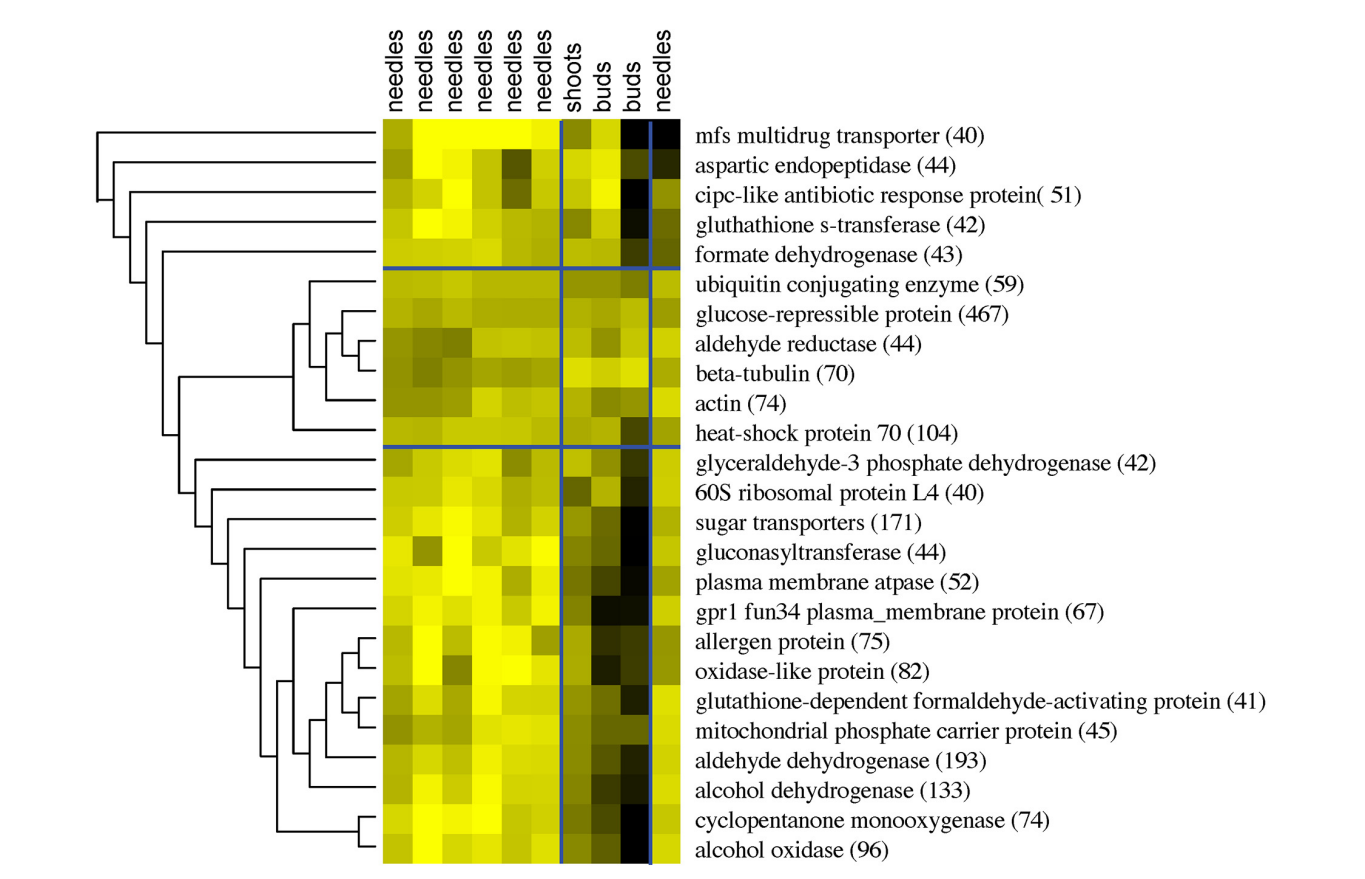
Heatmap representation of fungal expression patterns. Expression clustering based on FPKM values pooled across gene clusters, *i*.*e*. including both paralogs of gene families as well as species orthologs. The heatmap represents gene clusters with the highest numbers of sequences (>40).

When pooling the expression values of orthologous (clustered) sequences within a sample, we found that genes with housekeeping functions, such as actin, beta-tubulin, and ubiquitin conjugating enzymes, were expressed at almost constant levels across needle, bud, and vegetative shoot samples (Fig 2). Some high-copy number gene families also followed this pattern, including glucose-repressible proteins and heat shock proteins 70 (*HSP70*), while, by contrast, oxidases, oxygenases, dehydrogenases, and sugar transporters were expressed more highly in needles compared to the bud and vegetative shoot samples. Between the bud samples, major facilitator family drug transporters, aspartic endopeptidase, and CipC-like antibiotic response proteins were expressed at different levels (Fig 2).

## Conclusion

In conclusion, the results we presented in this study are consistent with previous findings [11–12], identifying a diverse and variable community of fungal endophytes and epiphytes living in Norway spruce and engaging in active metabolism. A notable characteristic of this work is that our results were purely serendipitous as our initial interest was only in the host plant transcripts. We unexpectedly obtained a rich and informative multi-use data set profiling both the host tree and associated microbial organisms suggesting that such an outcome should not be viewed negatively as “biological contamination” but as an exciting future opportunity to perform co-profiling when analysing deep coverage RNA-Seq data obtained from field-grown material.

## Supporting Information

S1 Fig Samples collected from Norway spruce. For each sample a brief description and sample ID are shown below a representative image of the associated plant tissue, while the sampling date is shown above.
(PNG)

S2 Fig Bioinformatics workflow of RNA data processing. We assembled reads from all samples into a single assembly (left column), computed Tau scores, GC content, and mapped the transcripts to the genome as well as to the Uniref90 protein database. For enriching for fungal transcripts (right column), we applied GC content and expression breadth filters to the reads and assembly respectively, clustered sequences by similarity, and performed functional annotation as well as phylogenetic analyses.
(PNG)

S3 Fig Putative taxonomic characterization of transcripts via protein alignments. Bar plot showing the number of transcripts by taxonomy (super)kingdoms. Parent summarises taxons hierarchically higher than the represented (super)kingdoms, NA summarises transcripts with no sequence similarity in the UniRef90 database. The number of transcripts is indicated at the top of every bar.
(PNG)

S4 Fig Taxonomic class and phylum of the fungal transcripts. **(a)** Number of transcripts per fungal phylum. The phylum are sorted by abundance top to bottom with Ascomycota (n = 81,181) and Basidiomycota (n = 4,839) being the most represented; the remaining phyla varying from n = 11 to n = 2. **(b)** A graph of the taxonomic hierarchy from species to phylum of the fungal transcripts, showing the broad species diversity of the largest clusters: Ascomycota (bottom) and Basidiomycota (top). **(c)** Similar to (a) for the fungal classes, with the Eurotiomycetes and Dothideomycetes classes being over-represented among the fungal transcripts. **(d)** Similar to (b) for the fungal classes (n = 24).
(PNG)

S5 Fig Characterisation of transcripts lacking taxonomic assignment by their GMAP alignments to the *P*. *abies* genome. **(a)** Boxplot of the tau scores for the no taxon transcripts split based on their GMAP alignments to the *P*. *abies* genome. The tau score ranges from 1 for complete specificity to 0 for equal expression in all samples. The transcripts having a GMAP alignment in the genome (99% of the GMAP hits cover 80% of the transcripts with at least a 90% identity) show a wide tau score distribution indicative of the presence of ubiquitously expressed transcripts as well as that of more tissue-specific transcripts. The transcripts having no GMAP alignment show a distribution typical of only tissue-specific expression (mean tau score of 0.98). **(b)** Percentage GC density distribution of the no taxon transcripts split based on their GMAP alignments to the *P*. *abies* genome. Transcripts having a GMAP alignment to the genome present a GC distribution typical of the *P*. *abies* transcripts. The transcripts without a GMAP alignment show a distribution enriched for higher percentage GC, similar to that of fungi. The shoulder observed under the peak of transcripts with GMAP alignments may indicate transcripts where the assembly contained gaps or created chimeras. **(c)** Scatterplot of log_2_ FPKM expression values *vs*. the percentage GC content for the transcripts with a GMAP alignment. Colouring indicates density, which is shaded from yellow (high) to blue (low). The expression of transcripts with a GMAP alignment resembles that of the Embryophita phylum. **(d)** Scatterplot of log_2_ FPKM expression values *vs*. the percentage GC content for transcripts with a GMAP alignment. Colouring as in (c). The expression of transcripts with no GMAP alignment resembled that of the fungal kingdom.
(PNG)

S6 Fig Phylogeny built on four nuclear genes. Shown are maximum-likelihood phylogenies based on fungal nucleotide sequences assembled from the spruce samples in context of known sequences, with highest sequence similarity to: **(a)** phosphoenolpyruvate carboxykinase; **(b)** NADP-dependent medium chain alcohol dehydrogenase; **(c)** beta lactamase; and **(d)** unspecific lipid transporter. Only branch with support values > 0.9 are shown. While clusters with more representative sequences yield better branch support (a, b), placement of clusters with fewer sequences is less certain (c, d). However, in all cases, at least one sequence is grouped with Dothideomycetes, and for (a,b) with Leotiomycetes.
(PNG)

S1 Table Sample IDs, description, and ENA submission IDs. Correspondence between the sample IDs as described in Nystedt *et al*., (2013), this manuscript and the ENA are shown in columns one to three. The fourth column contains a succinct description of the samples, refer to Nystedt *et al*., (2013) for full details.
(PDF)
