# Supplementary figures and images for: Serendipitous Meta-Transcriptomics: The Fungal Community of Norway Spruce (Picea abies)
Source: PLoS One. 2015 Sep 28;10(9):e0139080. doi: 10.1371/journal.pone.0139080 (PMC4586145; doi:10.1371/journal.pone.0139080)

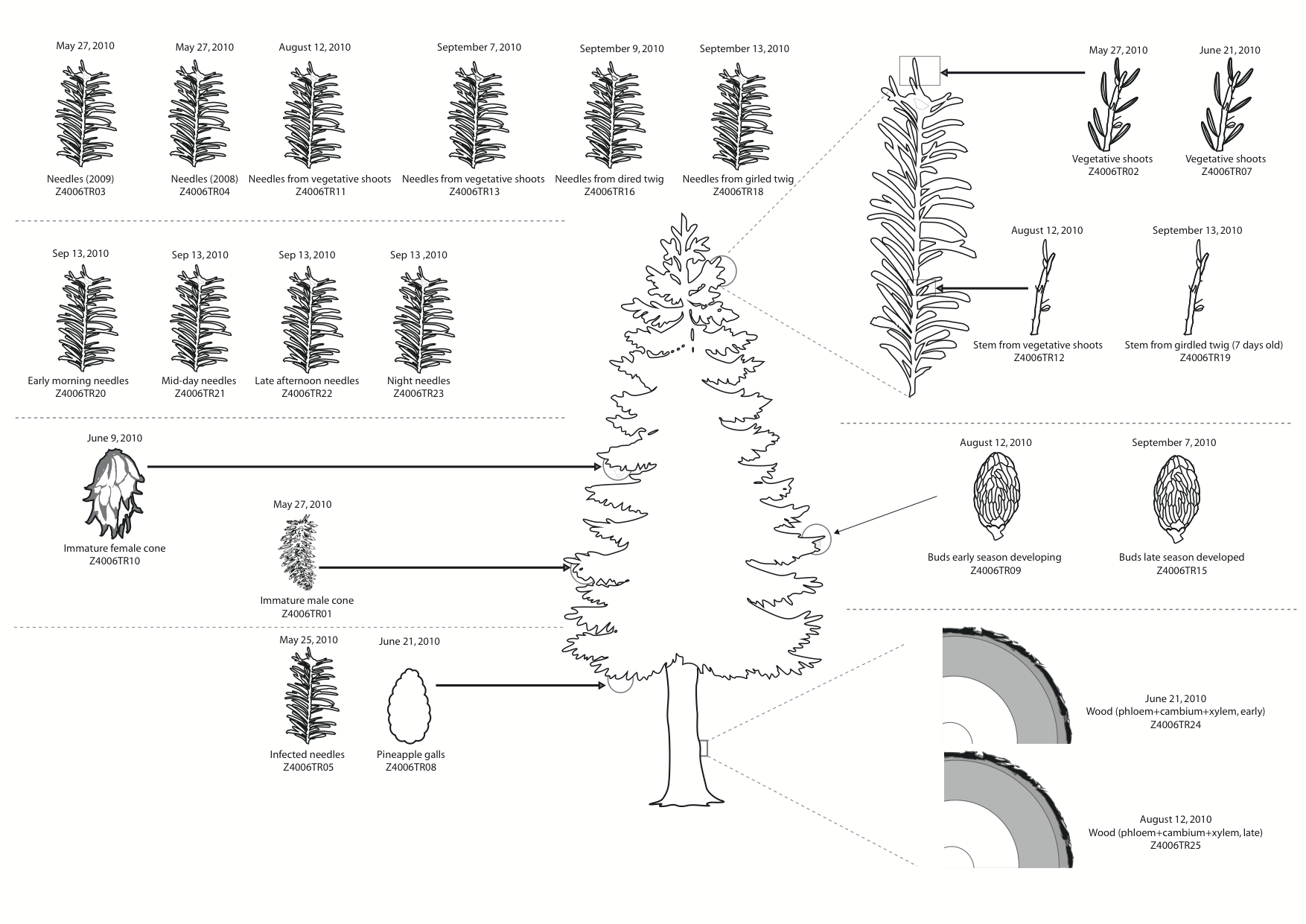

Supplement: S1 Fig — For each sample a brief description and sample ID are shown below a representative image of the associated plant tissue, while the sampling date is shown above. (PNG) [file pone.0139080.s001.png]

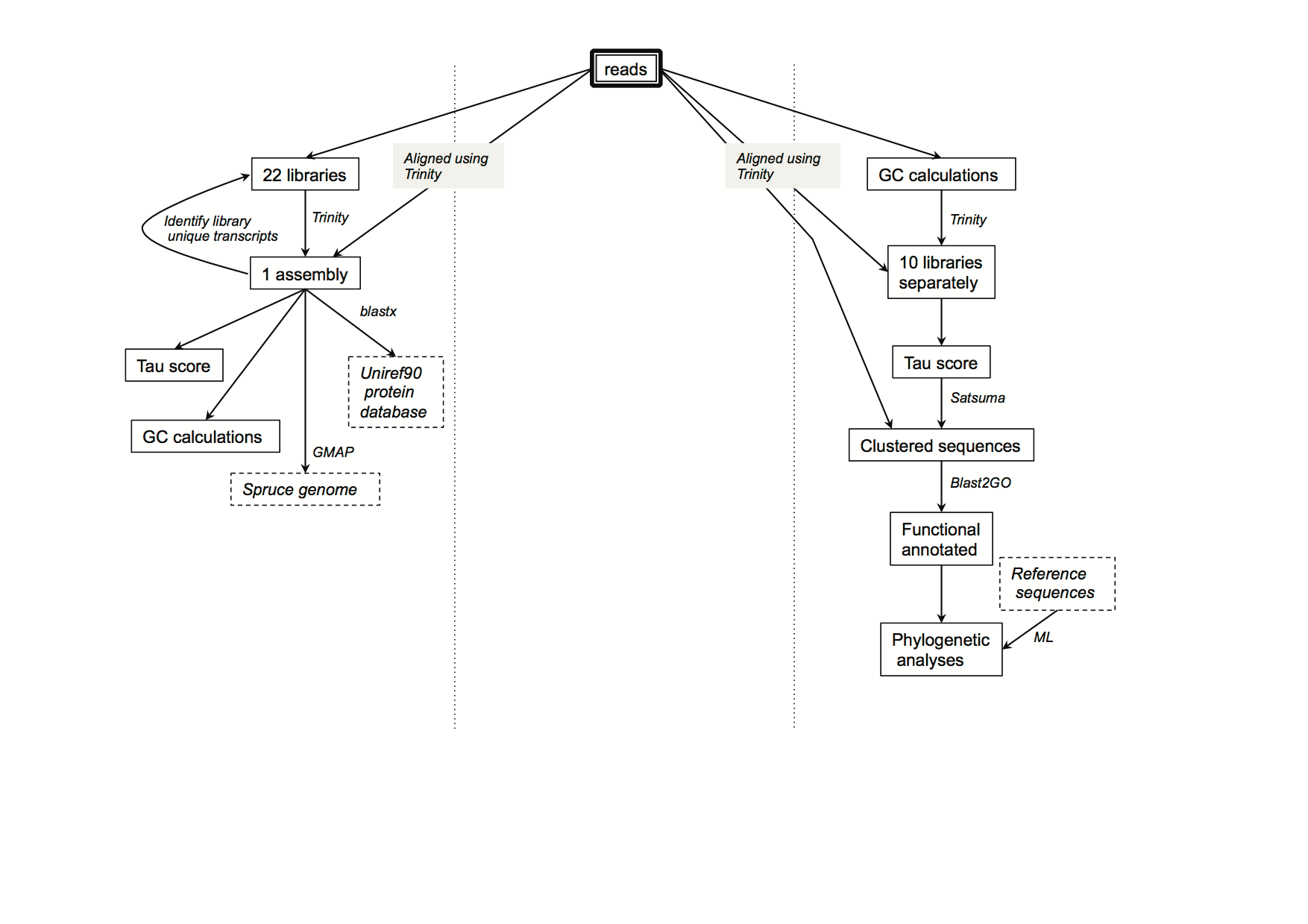

Supplement: S2 Fig — We assembled reads from all samples into a single assembly (left column), computed Tau scores, GC content, and mapped the transcripts to the genome as well as to the Uniref90 protein database. For enriching for fungal transcripts (right column), we applied GC content and expression breadth filters to the reads and assembly respectively, clustered sequences by similarity, and performed functional annotation as well as phylogenetic analyses. (PNG) [file pone.0139080.s002.png]

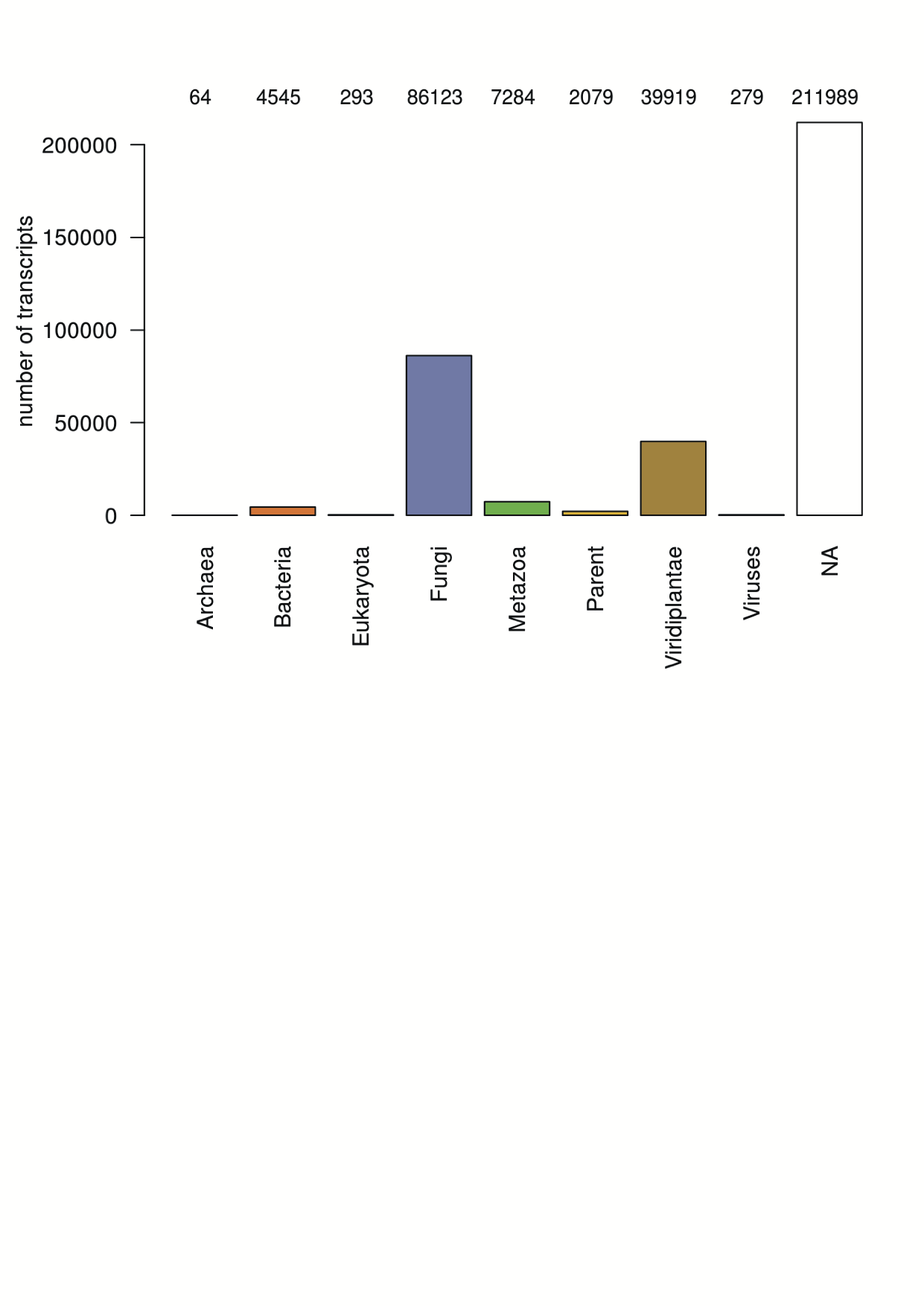

Supplement: S3 Fig — Bar plot showing the number of transcripts by taxonomy (super)kingdoms. Parent summarises taxons hierarchically higher than the represented (super)kingdoms, NA summarises transcripts with no sequence similarity in the UniRef90 database. The number of transcripts is indicated at the top of every bar. (PNG) [file pone.0139080.s003.png]

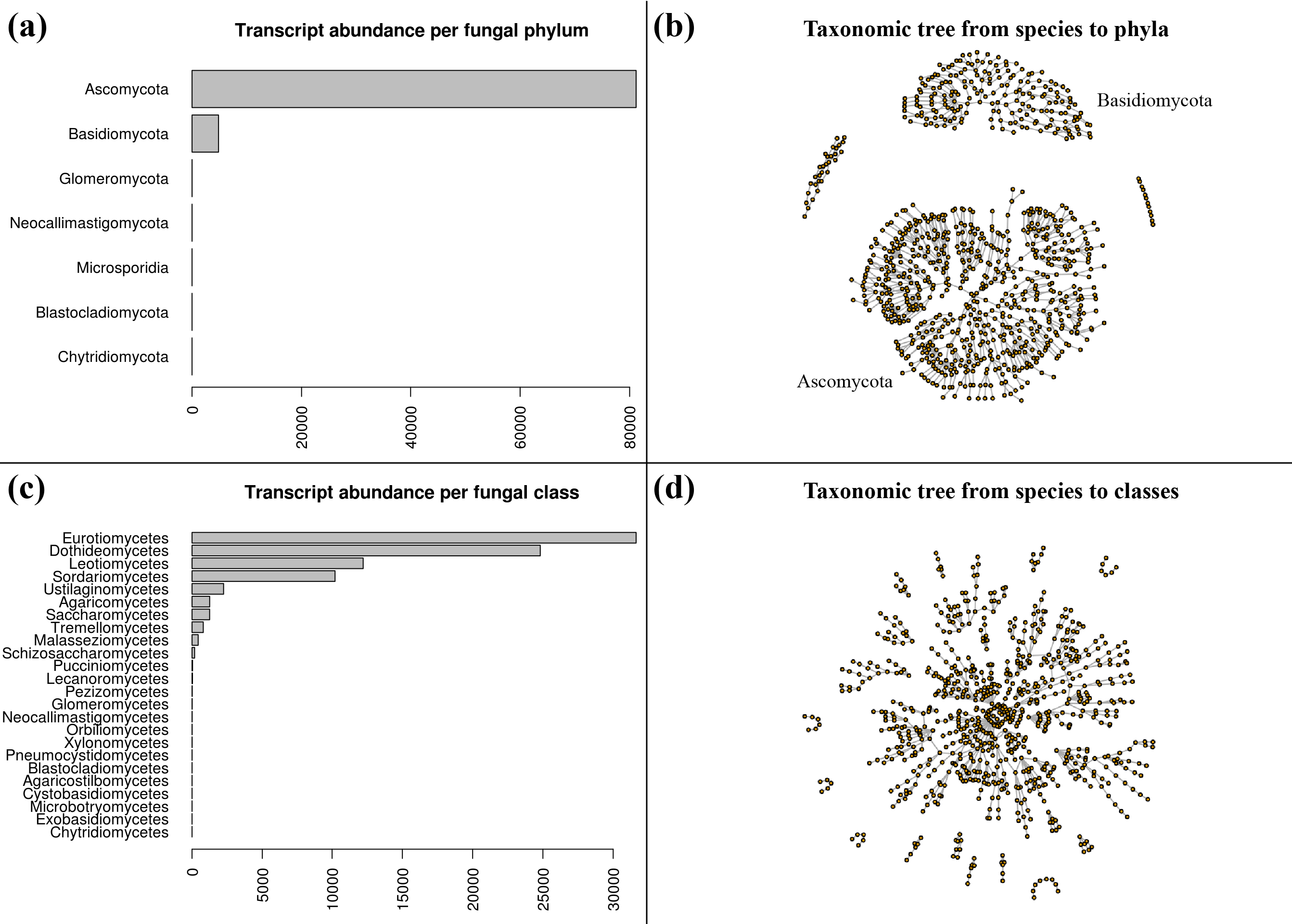

Supplement: S4 Fig — (a) Number of transcripts per fungal phylum. The phylum are sorted by abundance top to bottom with Ascomycota (n = 81,181) and Basidiomycota (n = 4,839) being the most represented; the remaining phyla varying from n = 11 to n = 2. (b) A graph of the taxonomic hierarchy from species to phylum of the fungal transcripts, showing the broad species diversity of the largest clusters: Ascomycota (bottom) and Basidiomycota (top). (c) Similar to (a) for the fungal classes, with the Eurotiomycetes and Dothideomycetes classes being over-represented among the fungal transcripts. (d) Similar to (b) for the fungal classes (n = 24). (PNG) [file pone.0139080.s004.png]

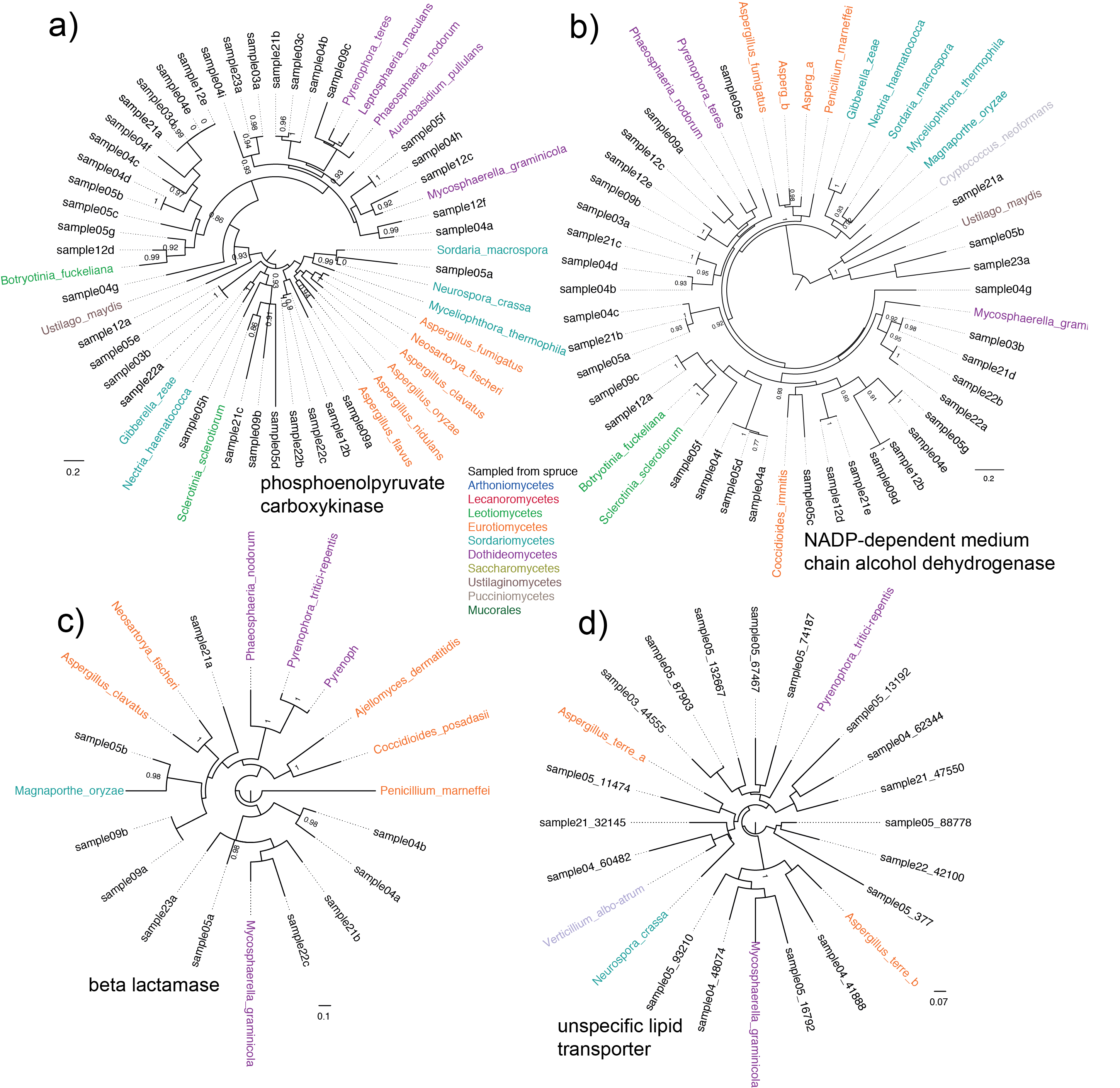

Supplement: S6 Fig — Shown are maximum-likelihood phylogenies based on fungal nucleotide sequences assembled from the spruce samples in context of known sequences, with highest sequence similarity to: (a) phosphoenolpyruvate carboxykinase; (b) NADP-dependent medium chain alcohol dehydrogenase; (c) beta lactamase; and (d) unspecific lipid transporter. Only branch with support values > 0.9 are shown. While clusters with more representative sequences yield better branch support (a, b), placement of clusters with fewer sequences is less certain (c, d). However, in all cases, at least one sequence is grouped with Dothideomycetes, and for (a,b) with Leotiomycetes. (PNG) [file pone.0139080.s006.png]
