# Supplementary material for: Serendipitous Meta-Transcriptomics: The Fungal Community of Norway Spruce (Picea abies)
Source: PLoS One. 2015 Sep 28;10(9):e0139080. doi: 10.1371/journal.pone.0139080 (PMC4586145; doi:10.1371/journal.pone.0139080)
Supplement: S1 Table — Correspondence between the sample IDs as described in Nystedt et al., (2013), this manuscript and the ENA are shown in columns one to three. The fourth column contains a succinct description of the samples, refer to Nystedt et al., (2013) for full details. (PDF) [file pone.0139080.s007.pdf]

Supporting Information Table S1 Sample IDs, descriptions and ENA accessions

Sample name	Sample ID	ENA ID	Sample description
Z4006TR01	Sample01	ERS235801	Male flower
Z4006TR02	Sample02	ERS235802	Vegetative shoots from 2010
Z4006TR03	Sample03	ERS235803	Needles from 2009
Z4006TR04	Sample04	ERS235804	Needles from 2008
Z4006TR05	Sample05	ERS235805	Infected needles from 2009
Z4006TR07	Sample07	ERS235806	Vegetative shoots from 2010
Z4006TR08	Sample08	ERS235807	Pineapple galls
Z4006TR09	Sample09	ERS235808	Buds, early season (Aug.)
Z3001TR10	Sample10	ERS235809	Female flower (cones)
Z4006TR11	Sample11	ERS235810	Needles from Vegetative shoots from 2010
Z4006TR12	Sample12	ERS235811	Stem from Vegetative shoots from 2010
Z4006TR13	Sample13	ERS235812	Needles from Vegetative shoots from 2010
Z4006TR15	Sample15	ERS235813	Buds, late season (Sept)
Z4006TR16	Sample16	ERS235814	Needles from dried twig (2 days at bench)
Z4006TR18	Sample18	ERS235815	Needles from girdled twig (1 week after girdle)
Z4006TR19	Sample19	ERS235816	Stem from girdled twig (1 week after girdle)
Z4006TR20	Sample20	ERS235817	Early morning (05:30, dawn) needles from 2010
Z4006TR21	Sample21	ERS235818	Mid-day (12:00) needles from 2010
Z4006TR22	Sample22	ERS235819	Late afternoon (19:30, dusk) needles from 2010
Z4006TR23	Sample23	ERS235820	Night (23:30) needles from 2010
Z4006TR24	Sample24	ERS235821	Wood (phloem, cambium, xylem) June 2010
Z4006TR25	Sample25	ERS235822	Wood (phloem, cambium, xylem) Aug. 2010

Correspondence between the sample IDs as described in Nystedt *et al.*, (2013), this manuscript and the ENA accessions are shown in columns one to three. The fourth column contains a succinct description of the samples, refer to Nystedt *et al.*, (2013) for full details.
